# The Efficacy of Percutaneous Vertebroplasty in Pain Relief in Patients with Pathological Vertebral Fractures due to Metastatic Spinal Tumors

**Published:** 2012-09-30

**Authors:** Mr Farrokhi, H Nouraei, A Kiani

**Affiliations:** 1 Associate Professor of Neurosurgery, Shiraz Neurosciences Research Center, Department of Neurosurgery, Shiraz University of Medical Sciences, Shiraz, Iran.; 2 Associate Professor of Orthopaedics, Shiraz Neurosciences Research Center, Department of Orthopaedic surgery, Shiraz University of Medical Sciences, Shiraz, Iran.; 3 Shiraz Neurosciences Research Center, Shiraz University of Medical Sciences, Shiraz, Iran.

**Keywords:** Polymethylmethacrylate (PMMA), Vertebroplasty, Spinal metastasis, Tumors, Spine, Pain

## Abstract

**Background:**

Metastatic spinal tumors are common and major causes of pathological spinal fractures that result in severe pain, weakness, and progressive neurological deficits. This study aims to evaluate the efficacy of percutaneous vertebroplasty (PVP) in pain-relief in patients with spinal fractures due to metastatic spinal tumors.

**Methods:**

We evaluated 25 documented cases of metastatic spinal tumors with pathologic vertebral fractures who were suffering from severe pain and underwent vertebroplasty. Degree of pain was measured by visual analog scale (VAS). The symptoms were evaluated 24 hours and 2 months after vertebroplasty regarding the degree of pain relief.Complications such as leakage, embolism and infection were assessed.

**Results:**

MeanVAS score was 8.23 before therapy in the patients that was reduced to 2.12 and 1 in the patients 24 hours and 2 months after vertebroplasty, respectively. The most common complication was cement leakage (44%) and there was no embolism or infection. Data was analyzed by SPSS version 18 software through ANOVA test with Greenhouse-Geisser correction and P-value of 0.00 was obtained in the patients 24 hours and 1 month after surgery.

**Conclusion:**

Considering significant decrease in the mean pain severity degree after the treatment, veretebroplasty seems to be significantly effective in pain relief in metastatic spinal tumors.

## Introduction

Spinal metastases are common and can lead to radiculopathy, myelopathy, deformity, severe pain and pathologic fracture that result in spinal cord compression (1). Spinal cord compressions present with pain in 90% of patients. Other neurological deficits may present that are less significant (2-4). spinal column with an incidence of 30% to 70% has the highest rate of metastatic neoplasms (5-7). Lung, prostate, breast, and renal malignancies are the major primary sites resulting in secondary spinal involvements (8,9). Most of the times first the posterior part of the vertebral body is invaded and then the other vertebral parts like anterior part, lamina and pedicles are involved (10).

Indications for surgical treatment include radiotherapy resistant disease, spinal cord compression, acute or progressive neurological deficits, previous radiotherapy of the spinal cord, spinal column instability, resistant pain despite previous therapies, and life expectancy of more than 3 months (11-13). Vertebral augmentation is now used in the management of pain in patients with spinal tumors (14). Vertebral augmentation techniques provide a minimally invasive alternative to open surgery in controlling pain due to pathologic compression fractures (14). Although the European experience with vertebroplasty in the setting of spinal metastases is more extensive (12,15-17), the indications for treatment among most North American series are currently heavily weighted toward osteoporotic bone disease (13,18-20). Surgical management generally involves vertebrectomy, reconstruction with polymethylmethacrylate (PMMA) bone cement, and stabilization with pedicle screws (18,21). The main goals of surgery are decompression of nerve roots, spinal cord, and reconstruction of the spinal column’s anatomy (22,23).

Percutaneous vertebroplasty (PVP) is a minimally invasive procedure in reducing pain caused by spinal compression fractures and improve vertebral column’s strength and mobility (24,25). Vertebroplasty is among the most commonly used treatments in spinal oncology for axial mechanical pain (14). In this type of treatment, radiopaque PMMA is injected in involved vertebra under fluoroscopic control (12-14) PMMA is composed of methylmethacrylate polymer as a powder and methylmethacrylate monomer as a liquid (26). Improvement of bone strength can be achieved even with minimal amounts of PMMA (27). The cement reinforces and stabilizes fractures and seems to alleviate pain (18,28).

Some complications such as PMMA leakage, rib fracture, spinal cord compression, infection, pneumothorax and cement embolism are reported to be associated with vertebroplasty. Most procedural complications are related to leakage of PMMA through cortical defects, with epidural compression of the neural elements; however most of these problems are clinically insignificant (29,30).

Pain is the most common symptom among patients with metastatic spinal tumors with spinal compression fractures (2-4). In this study, we evaluated the efficacy of vertebroplasty as a palliative treatment in such patients.

## Materials and Methods

In this prospective cohort study, we evaluated 25 documented cases of malignancies with secondary spinal involvement and vertebral body fractures at different sites. Our patients consisted of 11 males and 14 females with mean age of 53.5 (range 37 to 70 years). Severe pain was the main presenting symptom in these patients that had decreased their quality of life. The sites of pathologic fractures varied from T3 to L4 with major lumbar involvement and less thoracic fractures. All patients gave their informed consent in writing. Patients were evaluated by complete history, physical examination and radiological evaluation (X-ray, CT and MRI). The average pain was evaluated by using visual analogue scale (VAS) with scores ranging from 1 (no pain) to 10 (excruciating pain) before PVP. (31) The Vice-chancellor for research affairs of Shiraz University of Medical Sciences and Apadana Tajhizgostar Co. provided grant support but had no role in the design of the trial, the collection or analysis of data, or the preparation of the manuscript.

### Surgical technique

The patients were placed prone after induction of general anesthesia in operating room and single-plane C-arm equipment was used. Under strict sterile technique, the skin overlying the vertebral body to be injected was cleaned and draped. After a small skin incision, the disposable bone biopsy needle was inserted under fluoroscopic guidance and advanced until its tip reached the pedicle, then the needle was guided through the centre of the pedicle and into the vertebral body. A bilateral transpedicular approach was used only if there was inadequate instillation of cement with the unilateral approach under fluoroscopy. The PMMA bone cement was injected in different amounts from 3 to 6 mL considering the site and size of the fracture under lateral fluoroscopic control until the PMMA reached the posterior three-quarter of the vertebral body or the PMMA leaked into the disc space or paravertebral tissues (32). In cases of leakage pressure on the injecting syringe, it was released immediately and the injection was stopped for 2 to 3 minutes to allow the cement to harden and plug the leak, or for needle repositioning. Where PMMA did not enter the both sides of the vertebral body, the other pedicle was entered. At the completion of vertebroplasty, the needle was withdrawn, the puncture site closed with sterile strips, and a sterile dressing applied. Patients were kept in bed for a minimum of 1 hour to allow the cement to polymerize fully.

Open surgery was performed in 4 patients because of unstable vertebral fracture and deformity to keep spinal column’s strength and shape. The patients and their postoperative VAS pain scores were evaluated 24 hours after the surgery.

In order to make further evaluation, the degree of pain was also measured 2 months after the procedure.

Data was analyzed by SPSS version 18 software (SPSS Inc., Chicago, Illinois) through ANOVA test with Greenhouse- Geisser correction.

## Results

PVP was performed on 25 patients for a total of 25 procedures at 6 treated vertebral levels. The patients included 14 women and 11 men with a mean age of 53.5 years (range 37 to 70 years). The patients had pathologic fractures secondary to metastasis. Many patients had undergone previous therapy for spinal disease.

The sites of pathologic fractures varied from T3 to L4 with major lumbar involvement (72%) and less thoracic fractures (28%). The mean vertebral height lost before vertebroplasty was 7 mm. Four lumbar levels and 2 thoracic levels were treated. During the procedure, 6 mL cement was injected to 5 patients (8%), 5 mL was injected to 13 patients (52%), 4 mL was injected to 9 patients (36%), and 3 mL was injected to 1 patient (4%). 5 mL cement was injected to T4 (2%), 4 mL to T8 (3%), 4 mL to L1 (25%), 5 mL to L2 (15%), 5 mL to L3 (40%), and 4 mL to L4 (15%). The mean amount of cement injected per level in the patients was 4.5 mL and the total injection volumes ranged from 4 to 5 mL. The most vertebral involvement was L3. No infection or cement emboli occurred.

**Fig. 1:**
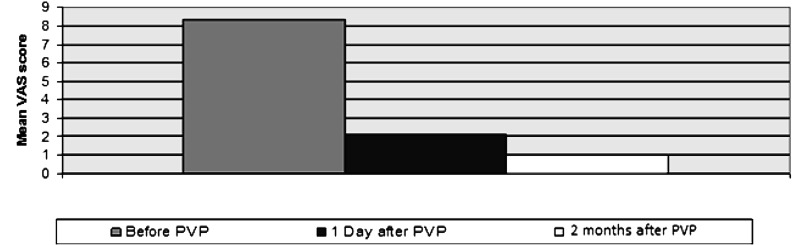
Mean VAS score of the patients before, 1 day and 2 months after PVP.

The original pain was improved. VAS scores of the patients were compared before and after the procedure and meaningful *P*-value of 0.00 was obtained 24 hours and 2 months after PVP (P≤0.05) that was considered statistically significant. Mean VAS pain degree of these patients was 8.23 before PVP that was decreased to 2.12 and 1 in 24 hours and 2 months afterwards (Fig. 1).

**Fig. 2:**
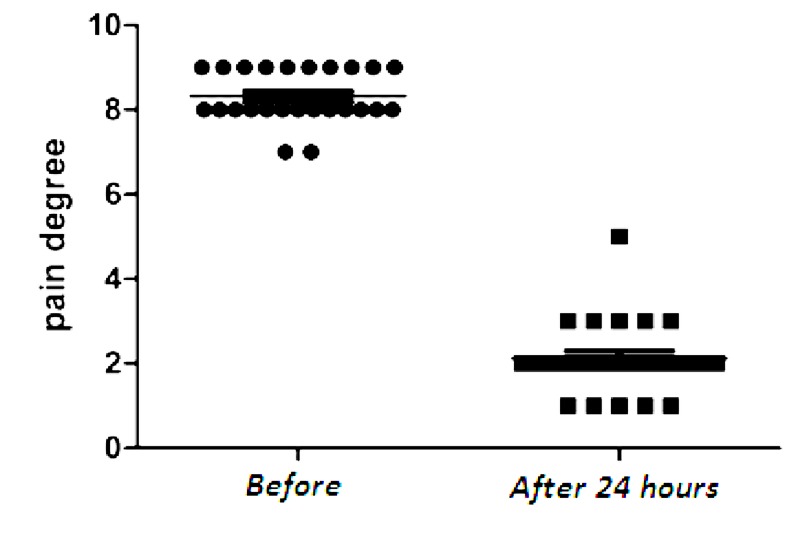
VAS score distribution of the patients before and 24 hours after PVP.

The patients experienced much less pain 24 hours after PVP with PMMA injection (Fig. 2) that continued decreasing gradually.

**Fig. 3:**
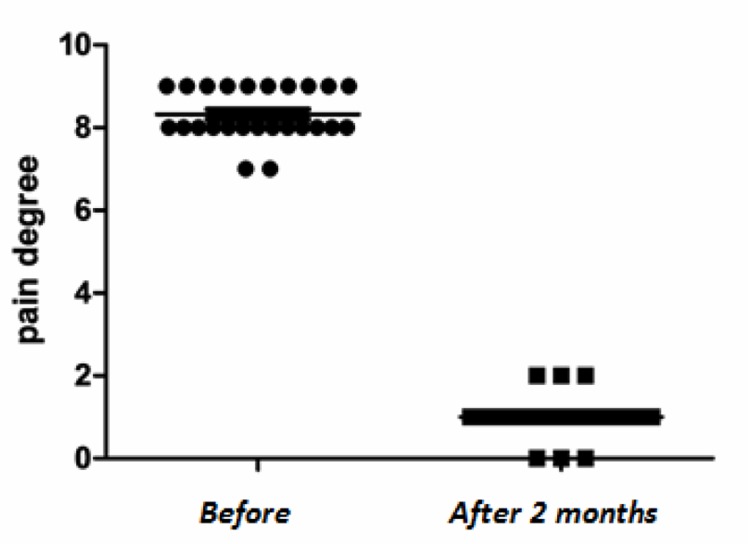
VAS score distribution of the patients before and 2 months after PVP.

After 2 months, 3 patients were symptom-free and the rest had a satisfactory pain degree (Fig. 3).

Histologic types of metastosis were breast carcinoma (56%), lung cancer (36%) and prostate (10%) in decreasing order. Leakage, infection and embolism as three major adverse effects of the procedure were assessed and 11patients with leakage including 5 paravertebral, 4 discal and 2 epidural leakages were reported (Table 1). There was no death related to PVP treatment. Extravasation sites include T4, T8, L1, L2, L3, and L4 levels. Cement leakage was noted in 6 levels (44%). Some levels had more than one type of cement extravasation. Leakage was noted on fluoroscopy during PMMA injection and CT scanning was performed immediately after the procedure. Cement extruded through a fractured endplate including the adjacent disc space (50%), paravertebral muscles (30%), anterior of vertebral body (20%), and epidural space (0). The extravasation was not appreciated because of the significantly increased bone density and difficulty of injecting into an osteoblastic metastasis. No neural foraminal extravasation of cement was noted. All cement leaks were asymptomatic. There were no radicular complaints as a result of cement extrusion. No neurological deficits resulting from vertebroplasty were detected. Radiography and MR imaging did not reveal any additional compression or change in the PMMA pattern. Plain radiography and CT scanning showed that the PMMA was in good position and there was no evidence of extravasation or dislodgment.

**Table 1:** Reported adverse effects in studied cases (N=25)

**Adverse ** **effect**	**n= Number of reported cases**	**Percentage of reported cases**
Leakage ( total )	11	44%
~ paravertebral	5	20%
~ discal	4	16%
~ epidural	2	8%
Infection	0	0%
Embolism	0	0%

## Discussion 

VAS scores obtained 24 hours and 2 months after PVP from our series of 25 patients showed significantly better pain relief in the patients with pathological vertebral fractures due to metastatic spinal tumors by PVP.

Medical therapy, surgery, and radiation are available treatments for metastatic diseases of spine. Surgical intervention is not usually the first line of therapy and mostly is used with goals of palliative pain control, neurologic function and spinal stability maintenance (33,34). Radiation is an effective therapy for radiation-sensitive tumors including prostate, hematopoietic and germ cell malignancies and can be successful in more than 80% of patients (2). While neurologic improvement and pain relief can be achieved with radiation in some patients (35,36). most surgeons only see patients after failure in their primary treatment and the vast majority of patients are sent directly to radiation oncology for conventional external therapy.

PVP is a safe, effective and minimally invasive surgical technique with decreased overall surgical complications which is successful at improving pain and contributes to spinal stabilization (37); it is a low-cost treatment with low morbidity in comparison to open surgery (14,38). Open surgery is another alternative, however it is associated with more complications, longer recovery period, high cost and also high morbidity. Hentschel et al (39).

Showed vertebroplasty is safe and effective in the setting of severe back pain caused by vertebral body fracture that is unresponsive to other therapies, even in the presence of relative contraindications to the procedures.

To date, percutaneous vertebral augmentation offers a minimally invasive approach for the treatment of pathologic vertebral compression fractures (40). PVP has become increasingly accepted as a treatment option in patients with intractable back pain due to vertebral compression fractures (41, 42). In our study, we found a statistically significant improvement in pain in the patients with pathological vertebral fractures due to metastatic spinal tumors by PVP. Our findings showed that the patients experienced much less pain 24 hours and 2 months after PVP with PMMA injection. Published results support the view that PVP is the treatment of choice in painful vertebral fractures refractory to medical management (38,43,44). Earlier reports found treatment with PVP to be rapidly effective and it might provide immediate pain relief in patients with pathological vertebral fractures (45). Cheung et al (25).

reported that PVP in metastatic fractures significantly decreased many patients’ back pain, reduced their intake of pain medications and was a safe procedure with no serious complications. Weill et al (17). reported that vertebroplasty of metastases is a minimally invasive procedure that provides immediate and long-term pain relief and contributes to spinal stabilization. Cotton et al (15). used PVP for metastases and reported that pain relief can occur despite insufficient lesion filling. Barr et al (20). reported that PVP provided significant pain relief in a high percentage of patients with osteoporotic fractures.

Some authors have correlated complications with excessive PMMA injection (16), whereas others have found no association (19). The most common complication in our study was cement leakage. Our study showed lower rate of cement leakage (44%) in vertebroplasty procedures compares favorably with published rates (15,19,20,46,47). Cement leakage is reported to occur during as many as 73% of vertebroplasty procedures (15). Sun et al. (48) reported that leakage of PMMA was detected in 64% treated vertebrae. Anselmetti et al (49). demonstrated that utilization of high-viscosity PMMA during routine PVP is safe and feasible and can significantly reduce venous cement leakage without any substantial changes in the vertebroplasty technique. No infection or cement emboli occurred in our study and no patients suffered neurological deficits resulting from vertebroplsty. In addition, no neural foraminal extravasation of cement was noted in our study.

## Conclusion

PVP significantly reduces the degree of pain in the patients with metastatic vertebral involvements.
